# Belowground responses to elevation in a changing cloud forest

**DOI:** 10.1002/ece3.2025

**Published:** 2016-02-24

**Authors:** Caitlin I. Looby, Mia R. Maltz, Kathleen K. Treseder

**Affiliations:** ^1^ Department of Ecology and Evolutionary BiologyUniversity of CaliforniaIrvineCalifornia92697

**Keywords:** Elevation gradient, fungal abundance, fungal community composition, microbial basal respiration, tropical montane cloud forests

## Abstract

Few studies have investigated how soil fungal communities respond to elevation, especially within TMCF (tropical montane cloud forests). We used an elevation gradient in a TMCF in Costa Rica to determine how soil properties, processes, and community composition of fungi change in response to elevation and across seasons. As elevation increased, soil temperature and soil pH decreased, while soil moisture and soil C:N ratios increased with elevation. Responses of these properties varied seasonally. Fungal abundance increased with elevation during wet and dry seasons. Fungal community composition shifted in response to elevation, and to a lesser extent by season. These shifts were accompanied by varying responses of important fungal functional groups during the wet season and the relative abundance of certain fungal phyla. We suggest that elevation and the responses of certain fungal functional groups may be structuring fungal communities along this elevation gradient. TMCF are ecosystems that are rapidly changing due to climate change. Our study suggests that these changes may affect how fungal communities are structured.

## Introduction

Tropical ecosystems have a disproportionate influence on global biodiversity, primary production, and biogeochemical cycling (Townsend et al. 2011). TMCF (Tropical montane cloud forests) are a distinct category of tropical ecosystem. They are high elevation forests characterized by persistent, low‐level cloud cover. These clouds alter light, temperature, and precipitation, thereby endowing TMCF with unique structural and functional characteristics (Bruijnzeel and Veneklaas 1998). For example, TCMF are shorter in stature and exhibit lower productivity, increased epiphytic biomass, and slower nutrient cycling rates compared to the lowland tropics (Grubb 1977; Tanner et al. 1998; Hobbie and Vitousek 2000).

Tropical montane cloud forests are extremely important hydrologically, biologically, and ecologically. In terms of hydrology, these ecosystems play a crucial role in local and regional water budgets and atmospheric circulation of water (Lawton et al. 2001; Nair et al. 2003; Ray et al. 2006). In terms of biology, TMCF are biodiversity hot spots. Even though they cover only 0.4% of the Earth's land surface, they contain 20% of the world's plant species and 16% of its vertebrate species (Myers et al. 2000).

In terms of ecology, TMCF have decreased rates of decomposition due to abiotic factors such as decreased temperatures and water‐logged soils (Vitousek and Sanford 1986). Inevitably, decreased rates of decomposition can lead to larger soil C stocks, higher soil C:N ratios, and stronger nutrient limitations present within TMCF (Vitousek et al. 1994; Raich et al. 2006; Soethe et al. 2008; Girardin et al. 2010; Moser et al. 2011; Dieleman et al. 2013; Whitaker et al. 2014a). This suite of characteristics, such as soil temperature, moisture, and C:N ratios can affect the composition of microbial communities within the soil.

Few studies have investigated microbial communities within TMCF, and information regarding soil fungal communities is especially limited. Fungal communities are sensitive to changes in temperature and precipitation (Rustad and Fernandez 1998; Schuur 2001; McGuire et al. 2011). In addition, soils with high C:N ratios tend to support a greater abundance of fungi (Fierer et al. 2009). Fungal communities are important because they conduct much of the decomposition that occurs within soils. Changes in the fungal community can induce changes in decomposition (Setälä and McLean 2004), ostensibly because fungal species vary in their role in decomposition (Hanson et al. 2008; McGuire et al. 2010). We know that abiotic factors, such as temperature and moisture, can cause slower rates of decomposition, but biotic factors such as shifts in fungal community might also contribute.

Soil temperature, moisture, and C:N ratios can shape fungal communities tend to shift predictably with elevation. However, shifts in belowground communities with elevation are variable (Bryant et al. 2008; Lin et al. 2010; Fierer et al. 2011; Singh et al. 2012, 2014; Meng et al. 2013; Whitaker et al. 2014b). This is especially true for fungi, and most fungal studies have focused on a specific functional group (e.g., Meier et al. 2010; Bahram et al. 2012; Gai et al. 2012; Gómez‐Hernández et al. 2012; Gorzelak et al. 2012; Zimmerman and Vitousek 2012; Coince et al. 2014).

Shifts in temperature and moisture along elevation gradients could elicit changes in the fungal community, which in turn can alter rates of decomposition and nutrient cycling along the gradient. Recently, Pellissier et al. (2014) used pyrosequencing to demonstrate that fungal communities varied across an elevation gradient in Swiss alpine grasslands. Within tropical montane forests, work has been conducted in Ecuador on mycorrhizal fungi (e.g., Camenzind and Rillig 2013; Camenzind et al. 2014, 2015). Moreover, Cantrell et al. (2013) used fatty acid composition and TRFLP analyses to determine how the abundances of microbial functional groups vary with elevation in Puerto Rico. Tedersoo et al. (2014) included TMCF in their global assessment of fungal diversity, but not along an elevation gradient. The current study is one of the first to examine how whole soil fungal communities respond to changes to elevation within TMCF using high‐throughput sequencing.

We used an elevation transect on the Pacific slope of the Cordillera de Tilarán in Monteverde, Costa Rica to determine how fungal and soil dynamics vary with elevation and season. We hypothesized that increasing elevation would be associated with increased soil C:N ratios due to decreased temperatures and availability of soil N. Furthermore, we hypothesized that fungal abundance should increase at higher elevations in response to higher soil C:N, which typically favors fungal growth. We also predicted decreased microbial basal respiration at higher elevations due to decreased temperatures and water‐saturated soils. Lastly, we hypothesized that fungal community composition would shift with elevation, with a decline in richness at higher elevations due to environmental filters (i.e., temperature and moisture) structuring these communities.

## Methods

### Field sites

An elevation transect was established in August 2013 along the Pacific slope of the Cordillera de Tilarán in the Monteverde Cloud Forest Reserve (10°18′N, 84°47′W) near Monteverde, Costa Rica. This study area is located on the leeward side of the mountain range in an undisturbed primary forest. The transect ranges from 1305 m.a.s.l. to 1850 m.a.s.l. with sites located every 50 m increase in elevation (Table S1). These 12 sites cover three Holdridge life zones: premontane, lower montane, and montane forests (Holdridge 1967; Table S1). The 50‐m intervals in elevation allowed us to examine high‐resolution changes in climate. We assessed differences in the dry season (January to May) versus the wet season (June to November). More detailed climatic and physical characteristics of the Monteverde Cloud Forest Reserve are provided by Clark et al. 2000.

### Soil sampling

We collected soils in August 2013 (wet season) and April 2014 (dry season). Average minimum and maximum daily temperatures for the 2013 wet season were 15.6°C and 20.3°C (measured at 10.3092° N, 84.8135° W; 1375 m.a.s.l.). Average monthly precipitation for the wet season was 2471 mm; 1795 mm was recorded during August 2013. Average minimum and maximum daily temperatures for the 2014 dry season were 14.9°C and 21.7°C. Average monthly precipitation was 183 mm during the dry season, with 385 mm recorded during April 2014. Soil sampling occurred at the end of the month for both seasons, and thus, precipitation measurements represent the month prior to sampling.

Twelve soil cores (2 cm diameter by 10 cm deep) were taken mainly in the O horizon at random locations along a 20 m line at each of the twelve sites; sampling locations were at least 2 m apart. Although we sampled mainly in the O horizon, we observed that the O horizon was a greater proportion of the sample with increasing elevation. A separate set of samples was collected at each site to determine soil bulk density. We used bulk density measurements to adjust values to a square meter basis for microbial basal respiration and fungal abundance in order to understand potential ecosystem‐level effects. We transported soil on ice to UC Irvine within 48 h of collection. Prior to analysis, soil from each site was composited, homogenized, and sieved at 2 mm. Soils were stored at 18°C for biogeochemical analyses and were processed within 72 h after collection. Soils were stored at −20°C for fungal community analysis.

### Soil properties

Soil temperature was measured at 10 cm depth in four randomly selected locations at each site. Subsamples were weighed, dried 65°C for 48 h, and then re‐weighed to determine gravimetric moisture content. Soil pH was determined using a 1:2 ratio (w/v) of soil to DI H_2_O. Soil C and N concentrations were measured by combustion on an elemental analyzer (Flash EA 1112, Thermo Scientific, Waltham, MA) and used to determine C:N ratios.

### Fungal abundance

Fungal hyphal length was measured as a metric for total soil fungal abundance. We measured the length of fungal hyphae during the wet and dry seasons using a modified procedure from Brundrett et al. (1996). Briefly, three subsamples of 4 g (wet weight) soil were extracted with 1.5 mol L^−1^ solution of sodium hexametaphosphate. This soil solution was passed through a 0.2‐*μ*m nylon filter to collect the hyphae. Filters were stained with acid fuchsin, mounted on a glass slide with PVLG (polyvinyl lactic acid) slide mounting medium, and dried at 65°C overnight. Hyphal lengths were measured using a gridline intersect method at 200× on a Nikon phase‐contrast microscope (Nikon Eclipse e400, AG Heinze, Lake Forest, CA).

### Microbial basal respiration

Microbial basal respiration is the rate of CO_2_ respiration in soil originating from the decomposition of organic matter by microbes (Alef and Nannipieri 1995). Microbial basal respiration was measured in a laboratory incubation using an infrared gas analyzer (PP Systems EGM‐4, Amesbury, MA) to monitor CO_2_ flux using a closed chamber approach. Measurements were made at 20°C during a 4‐h incubation, which provides the best prediction for initial rates of respiration (Creamer et al. 2014).

### Fungal community composition

We extracted DNA from 3 ~0.25 g samples of soil from each elevation using the MoBio PowerSoil kit (MoBio, Carlsbad, CA). DNA concentrations were standardized to 10 ng/*μ*L prior to PCR amplification.

Primers targeting the 5.8S encoding gene were modified to amplify the ITS2 region of the ribosomal encoding genes from fungi. By producing a shorter amplicon than primers targeting the entire ITS region, these primers reduce species bias and PCR chimeras while maintaining the same level of fungal diversity (Ihrmark et al. 2012). A ~340 bp region of fungal ITS DNA was amplified with a staggered primer design. This included a forward primer (ITS9f; AATGATACGGCGACCACCGAGATCTACAC TCTTTCCCTACA CGACGCTCTTCCGATCT NNNNNGAACGCAGCRAAIIGYGA) and barcoded, reverse primers that contain the reverse complement of the 3' Illumina adapter (CAAGCAGAAGACGGCATACGAGAT), a unique, 12 bp barcode, a pad (AGTCAGTCAG), a linker sequence (CC), and the ITS4 primer (TCCTCCGCTTATTGATATGC). The staggered design incorporated an additional 0, 1, 2, or 3 bp preceding the ITS4 primer (i.e., CC‐ITS4, CC‐G‐ITS4, CC‐AG‐ITS4, or CC‐CAG‐ITS4). This staggered design increases the level of diversity of amplicon sequences across the Illumina Miseq flowcell early in the read, which improves the accuracy of amplicon cluster detection and resolution and overall sequence quality (Tremblay et al. 2015).

Each reaction contained: 21.5 *μ*L of Platinum PCR Supermix (Invitrogen, Carlsbad, CA), 0.75 *μ*L of each primer (10 *μ*mol L^−1^), 1 *μ*L of BSA (10 mg mL^−1^), and 1 *μ*L of (10 ng) of DNA. The reactions ran with a hot start at 94°C for 5 min, 35 cycles of 95°C for 45 sec, 50°C for 1 min, 72°C for 90 sec, and a final extension step of 72°C for 10 min. PCRs from each sample were run in triplicate, pooled, and purified with Agencourt AMPure XP magnetic beads (Beckman Coulter, Brea, CA). Purified samples were quantified with the Qubit dsDNA High Sensitivity Assay Kit (Life Technologies, Grand Island, NY) and pooled in equimolar concentrations. The pooled sample was sequenced as 2 × 300 bp paired end reads on one lane of an Illumina MiSeq sequencer at the Genomics core in the Institute for the Integrative Genome Biology at the University of California, Riverside.

We obtained a total of ~16.9 million sequences, which were processed through QIIME (Quantitative Insights Into Microbial Ecology) pipeline v. 1.9.0 (Caporaso et al. 2010). In QIIME, sequences were quality checked, aligned, and clustered into OTUs (operational taxonomic units) at a 97% similarity cutoff. After quality control, our dataset contained ~3.8 million high‐quality sequences that were used to generate the OTU table. One representative sequence from each OTU was chosen, and the closest taxonomic identity was determined via BLAST comparison in GenBank and the UNITE (v.7; release date 3.2.2015) database. All sequences were deposited in the GenBank with the accession number SH200172.07. To avoid bias due to different library sizes, samples were rarefied to the lowest coverage: 14,628 sequences per sample. Dataset wide singletons and nonfungal OTUs were identified and discarded manually prior to statistical analysis.

Finally, we assigned OTUs to functional groups based on taxonomic identity. We used the same designations as (Tedersoo et al. 2014)**,** although we grouped all pathogenic and parasitic taxa into one category, “pathogens,” regardless of host. We also split saprotrophic taxa into “free‐living filamentous fungi” and “yeasts” depending on morphotype. Yeasts included facultative and obligate yeast. We were able to classify 43–61% of OTUs to functional group within each sample.

### Statistical analyses

We used linear regressions to test for relationships between the different soil parameters and elevation. We nested subsamples within elevation to account for variances that may be associated in our experimental design. Differences are reported as significant when *P *<* *0.050. Data for microbial basal respiration and relative abundances of Chytridiomycota and Zygomycota were ranked, and analyses were performed to ensure that outliers were not solely driving these relationships. In order to examine differences between these relationships during the wet and dry seasons, an ANCOVA (analysis of covariance) was used (Sokal and Rohlf 2012). Linear regressions are reported as different when *P *<* *0.050 indicating that there are differences in seasonality in the relationships between the measured soil parameter and elevation. A multiple regression analysis was conducted on dry season data to test for the relationship between hyphal length and soil moisture versus microbial basal respiration. All statistical analyses were carried out using the statistical program R (Version 0.98.507, R Development Core Team 2013).

Taxonomic richness (alpha diversity) was computed using observed number of fungal OTUs, Shannon diversity index, and Simpson's diversity index in QIIME. Relative abundances of fungal phyla were calculated as the proportion of sequences present in each sample. We determined these proportions by calculating the total number of sequences from each phylum within that subsample and dividing by the total number of sequences per subsample. For taxonomic richness of fungal communities and relative abundance of fungal phyla, we used linear regressions and ANCOVA analyses to test for differences between elevations and seasons. To determine the relationship between fungal community composition and elevation, we used a PERMANOVA analysis using Bray–Curtis dissimilarity and the adonis function in the Vegan package of R (Anderson 2001; Oksanen et al. 2012). In this model, we also tested the relationships between fungal community composition and season, Holdridge life zone, soil temperature, soil moisture content, C:N ratios, and pH independently. NMS (Nonmetric multidimensional scaling) plots were used to visualize fungal community composition.

**Figure 1:**
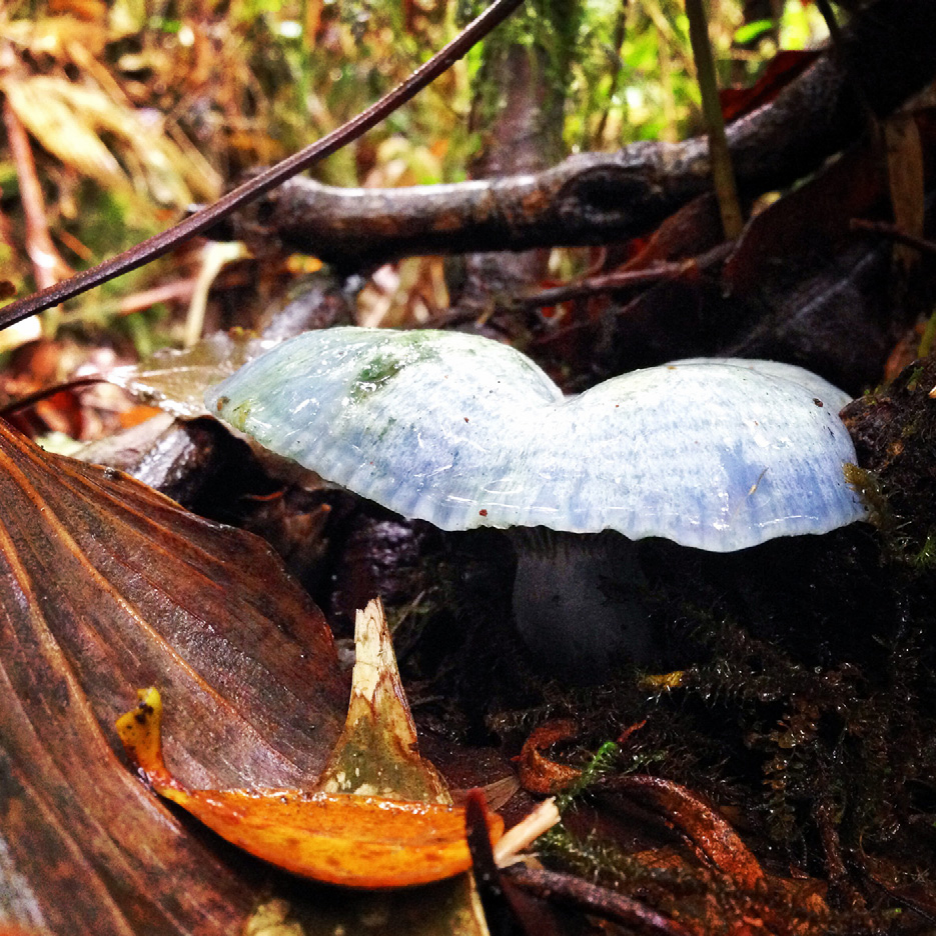
*Lactarius indigo* (Russulaceae) along the elevation transect established in the Monteverde Cloud Forest Reserve (10°18′N, 84°47′W).

To examine changes in functional groups with elevation, we counted the number of OTUs (i.e., richness) within each functional group in each sample. We then took the average of the three replicates per site/season and performed linear regressions (Fig. 1).

## Results

### Soil properties

**Figure 2:**
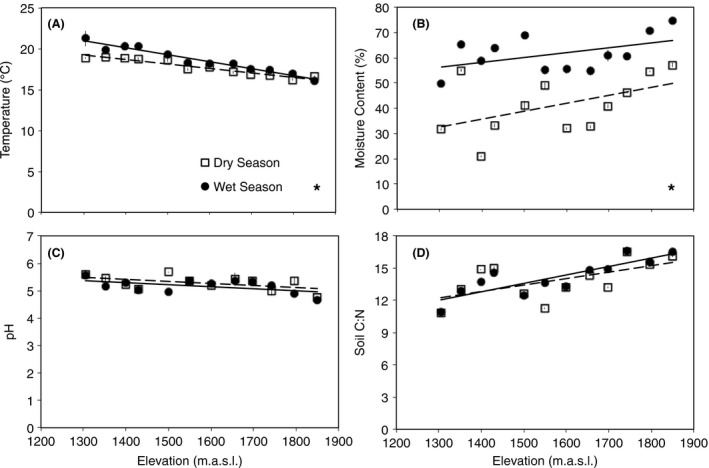
Elevational trends were found in (A) soil temperature, (B) soil moisture content, (C) soil pH, and (D) soil C:N ratios during both the wet (●) and dry (□) seasons. Lines are significant best‐fit regressions for wet (solid line) and dry (dashed line) seasons. Symbols are mean ± SE (*n *=* *3) for each site. Significant differences between seasons are designated with an asterisk. Statistical results are presented in Table S2.

Soil temperature decreased significantly with elevation (*P *<* *0.001 for each season; Fig. 2A; Table S2), and the extent of the decline differed between seasons (*P *<* *0.001). More specifically, soil temperature declined about 5°C during the wet season, but only 3°C during the dry season.

In addition, soil moisture increased significantly with elevation (*P* (wet) = 0.008, *P* (dry) = 0.003; Fig. 2B; Table S2). As expected, soil moisture was higher during the wet season than the dry season, and there was a significant difference between the wet and dry seasons (*P *<* *0.001).

Soil pH decreased significantly with elevation during each season (*P* (wet) = 0.001, (*P* (dry) = 0.011; Fig. 2C; Table S2), and the seasons did not differ significantly from one another (*P *=* *0.345).

Moreover, soil C:N ratios increased significantly with elevation during both seasons (*P *<* *0.001 for each season; Fig. 2D; Table S2) with no significant differences between season (*P *=* *0.462).

### Fungal abundance

**Figure 3:**
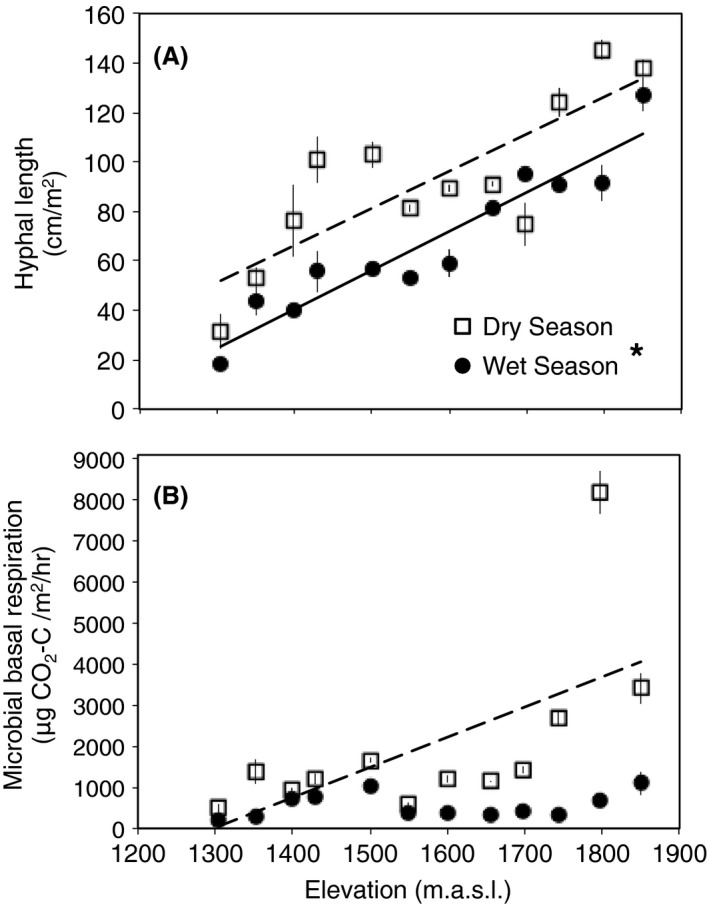
Elevational trends were found in (A) fungal abundance during the dry (□) and wet (●) seasons and (B) microbial basal respiration increased during the dry season. Seasonality had a significant effect on the relationships between fungal abundance and elevation. Lines are significant best‐fit regressions for wet (solid line) and dry (dashed line) seasons. Symbols represent means ± SE (*n *=* *3). Significant differences between seasons are designated with an asterisk. Statistical results are presented in Table S2. Microbial basal respiration data were ranked to ensure that outliers were not solely driving the relationship.

Fungal hyphal length increased significantly with elevation during the wet and dry seasons (*P *<* *0.001 for each season; Fig. 3A; Table S2), and there was a significant difference between the seasons (*P *<* *0.001). There was a stronger correlation between hyphal length and elevation during the wet season (*r* ^2^ = 0.862) than the dry season (*r* ^2^ = 0.637).

### Microbial basal respiration

Rates of microbial basal respiration increased significantly with elevation during the dry season (*P *<* *0.001; Fig. 3B; Table S2). Seasonality was most pronounced in the montane forest sites (1743–1850 m.a.s.l.), where microbial basal respiration was notably higher during the dry season than in the wet season. To determine a potential cause of this trend, we performed a further analysis to quantify relationships between this parameter and fungal abundance and soil moisture content during the dry season. Fungal abundance and soil moisture both had a significant effect on microbial basal respiration (*r* ^2^ = 0.559). Microbial basal respiration increased significantly with increasing fungal abundance (*P *<* *0.001) and, to a lesser extent, increasing soil moisture (*P *=* *0.022). Dry season data were used for both parameters due to the fact that the trend was only present during the dry season.

### Fungal diversity and community composition

**Figure 4:**
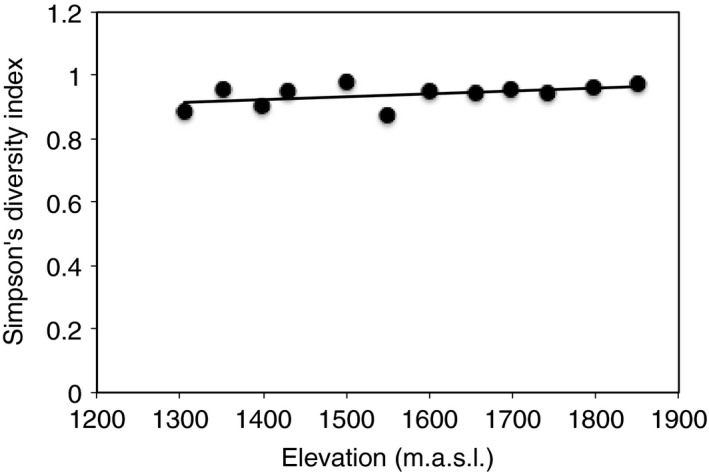
Fungal taxonomic richness decreases with elevation during the wet season according to the Simpson's diversity index. Values for Simpson's diversity index are inversely proportional to diversity: higher values indicate decreased diversity. Lines are significant best‐fit regression, and symbols represent means ± SE (*n *=* *3). Table S3 shows other diversity metrics and associated statistical results.

Taxonomic richness decreased significantly with elevation during the wet season, based on Simpson's diversity index (*P *=* *0.008; Fig. 4; Table S3) and observed number of fungal OTUs (*P *=* *0.050; Table S3). Values for Simpson's diversity index are inversely proportional to diversity: higher values indicate decreased diversity. The Shannon diversity index did not vary significantly with elevation. In addition, there were no significant relationships of any of the diversity metrics with elevation during the dry season (Table S3).

**Figure 5:**
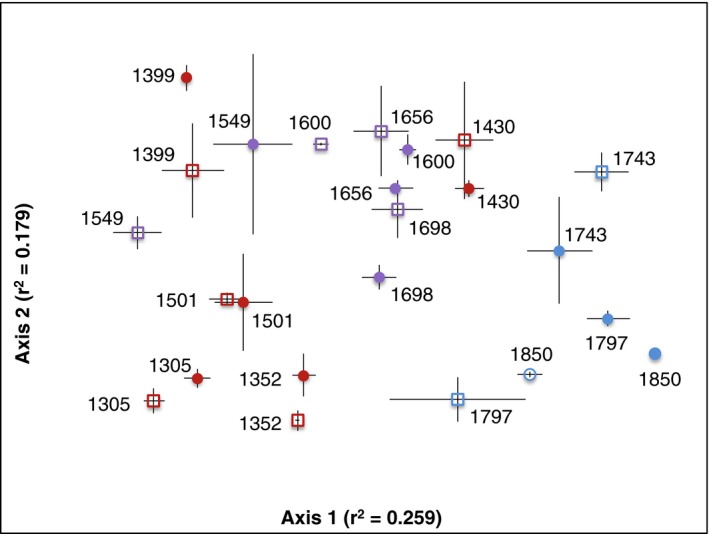
Compositional differences in fungal communities with elevation, as a NMS (nonmetric multidimensional scaling) plot. Fungal communities differed significantly with elevation (Table 1); *r* ^2^ values represent the correlation between the ordination axes and our data. Numbers adjacent to symbols indicate elevation. Open squares signify dry season samples, and closed circles signify wet season samples. Symbols are means ± SE of three replicates. Data are colored by life zone, including premontane forest (red), lower montane forest (purple), and montane forest (blue) representing low, mid‐, and higher elevation sites.

Fungal communities shifted significantly with elevation (*P *<* *0.001; Fig. 5; Table 1) and with other soil conditions that co‐varied with elevation: temperature, moisture, C:N, and pH (*P *<* *0.001 in each case). Variations in fungal communities were also explained by Holdridge life zone (*P *<* *0.001) and, to a lesser extent, by season (*P *=* *0.039).

**Table 1:** PERMANOVA results for fungal community composition as a function of elevation, season, Holdridge life zone, and soil properties^a^

Environmental parameter	*F*	*r* ^2^	*P*
Elevation	7.34	0.095	**<0.001**
Season	1.74	0.024	**0.039**
Holdridge life zone	5.94	0.146	**<0.001**
Temperature	4.37	0.058	**<0.001**
Soil moisture content	3.91	0.059	**<0.001**
C:N	9.19	0.116	**<0.001**
pH	4.55	0.061	**<0.001**

a Significant *P*‐values in bold.

**Figure 6:**
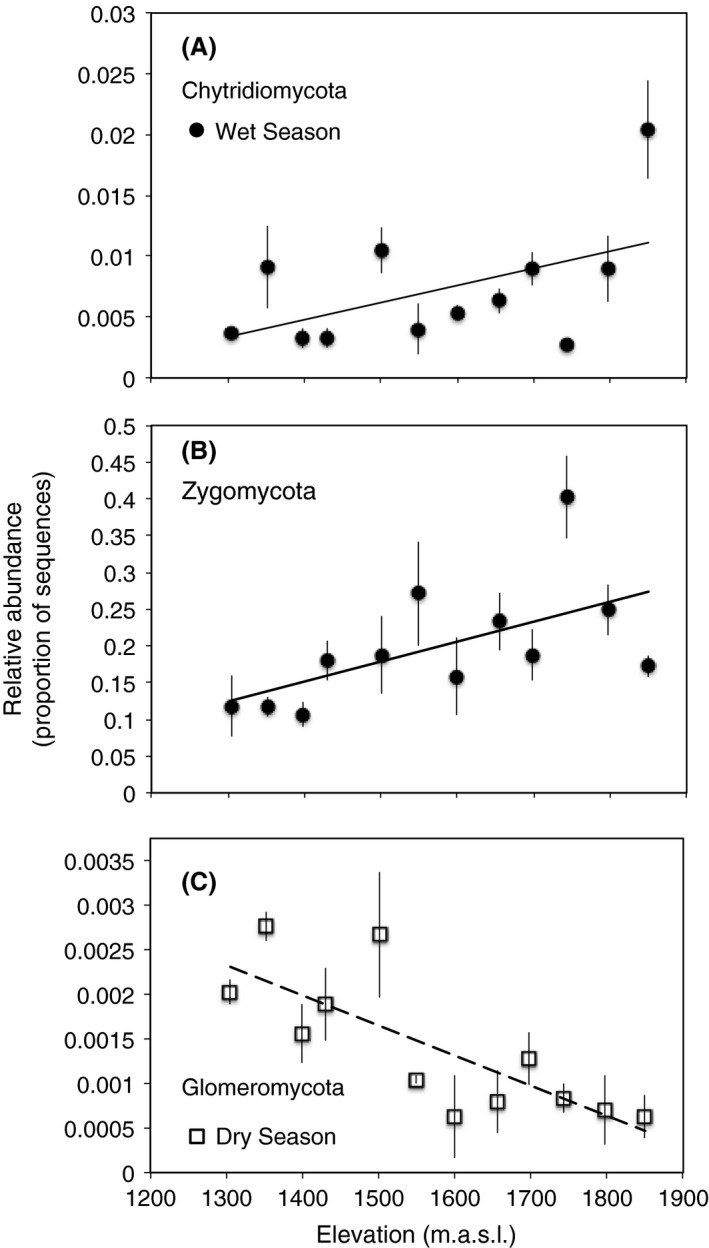
Relative abundance of (A) Chytridiomycota and (B) Zygomycota with elevation during the wet (●) season and (C) Glomeromycota during the dry (□) season. Lines are significant best‐fit regressions for wet (solid line) or dry (dashed line). Symbols represent mean ± SE (*n *=* *3). Chytridiomycota and Zygomycota data were ranked to ensure that outliers were not solely driving relationships. Relative abundance of other phyla and statistical results are presented in Table S4.

**Figure 7:**
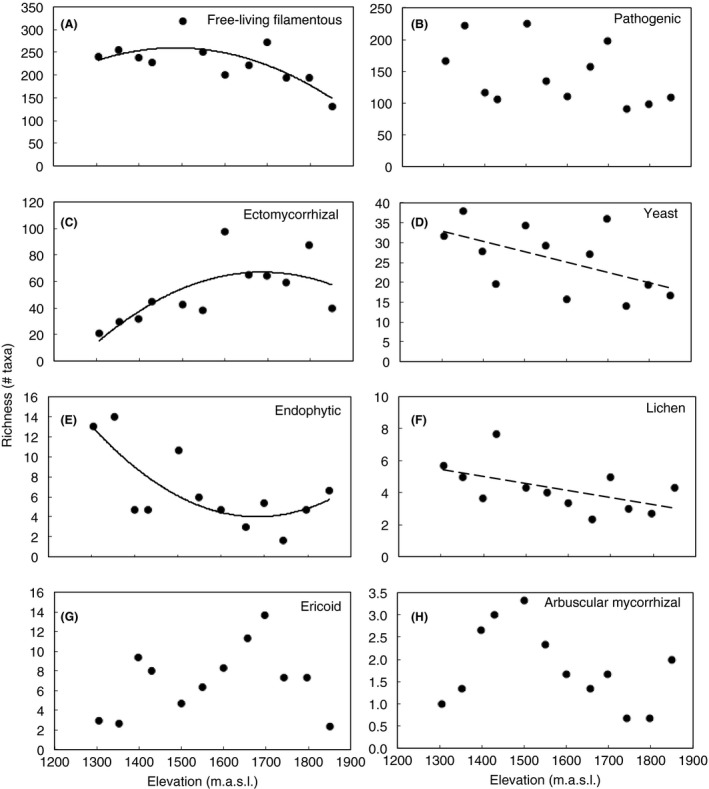
Taxonomic richness of functional groups by elevation during the wet season. Fungal taxa were grouped as (A) free‐living filamentous, (B) pathogenic, (C) ectomycorrhizal, (D) yeast, (E) endophytic, (F) lichen, (G) ericoid, or (H) arbuscular mycorrhizal. Lines are best‐fit regressions, and symbols represent number of taxa found at each elevation. Statistical results are presented in Table S5.

Shifts in fungal community composition were associated with shifts in the taxonomic richness within certain fungal phyla. During the wet season, the proportion of sequences representing the phyla Chytridiomycota and Zygomycota increased with elevation (*P* (Chytridiomycota) = 0.018; Fig. 6A; *P* (Zygomycota) = 0.003; Fig. 6B; Table S4). In contrast, the Glomeromycota decreased with elevation (*P *=* *0.001; Fig 6C; Table S4) during the dry season. The taxonomic richness of some functional groups varied significantly with elevation during the wet season (Fig. 7; Table S5). Specifically, OTU richness of free‐living filamentous fungi declined at the highest elevations (*P *=* *0.005; Fig. 7A). Endophytic OTU richness declined markedly with elevation in the lower half of the gradient and remained consistently low in the upper half (*P *=* *0.005; Fig. 7E). ECM (Ectomycorrhizal) fungi displayed the opposite trend – OTU richness increased with elevation, leveling off at the highest sites (*P *=* *0.006; Fig. 7C). Yeasts and lichens declined linearly with elevation, but only marginally significantly (*P* (yeasts) = 0.060, *P* (lichens) = 0.071; Fig. 7D and F). There were no elevational trends in OTU richness in pathogenic, ericoid, and arbuscular mycorrhizal functional groups in the wet season. Moreover, the only functional group that varied significantly with elevation during the dry season was arbuscular mycorrhizal fungi (i.e., Glomeromycota; Fig. 6C).

## Discussion

### Abiotic factors

Elevation gradients can be used to understand the influence of important abiotic factors on belowground communities and associated processes (Malhi et al. 2010). Our study provided an examination of the responses of soil dynamics and fungal communities to changes in elevation. Soils at higher elevations experience lower temperatures and wetter conditions. Results from this study support the notion that there may be abiotic and biotic filtering on belowground communities.

We found that soil C:N ratios increased with increasing elevation. Many elevation studies have reported higher C:N ratios and larger pools of C in soils at higher elevations (Raich et al. 2006; Soethe et al. 2008; Girardin et al. 2010; Moser et al. 2011; Dieleman et al. 2013; Whitaker et al. 2014a). Moreover, tropical montane forests can be N limited (Vitousek and Sanford 1986; LeBauer and Treseder 2008; Fisher et al. 2013). Although we did not directly test for N limitation, the increase in C:N ratios with elevation suggests low N availability higher elevations.

### Fungal abundance

Elevated soil C:N ratios at the higher elevation sites may have contributed to the observed increases in fungal abundance. Indeed, high soil C:N is often associated with fungal‐dominated communities (Fierer et al. 2009). A previous study found no relationship between hyphal respiration and elevation (Fisher et al. 2013). In an elevation gradient in Andean Peru, Whitaker et al. (2014a) likewise found an increase in fungal dominance as well as soil C:N ratios at higher elevations. Nevertheless, few other studies have directly assessed fungal abundance within tropical montane forests. Litter at higher elevations is typically more recalcitrant due to increased nutrient limitation, leaf thickness, and sclerophylly (Bruijnzeel and Veneklaas 1998). This pattern could favor the presence of fungi, which can break down more recalcitrant organic matter than many bacteria (de Boer et al. 2006; Floudas et al. 2012; Schneider et al. 2012). Higher amounts of organic matter and typical of higher elevations (Grieve et al. 1990; Tanner et al. 1998) can also support a greater abundance of fungi.

Our results demonstrate that fungal abundance was greater in the dry season than in the wet season. Other studies have shown increased fungal abundance during drier conditions (Sigüenza et al. 1996; Guadarrama and Álvarez‐Sánchez 1999; Hawkes et al. 2011). This could be due to plant phenology. During the dry season, there is increased litterfall, providing more substrate for fungi to decompose. Alternately, fungal turnover may have been slower in these drier conditions.

### Microbial activity

The increase in fungi could have contributed to the rise in microbial basal respiration with elevation during the dry season. Other studies have found similar trends with elevation. In a cloud forest in Colombia, Cavelier and Peñuela (1990) found that increased soil respiration accompanied increased moisture at higher elevations. In the current study, microbial basal respiration was correlated with soil moisture as well as fungal abundance. It is possible that the greater abundance of fungi at higher elevations – and during the dry season – improved the potential for mineralization of organic material. This potential was then modified by water availability, so that microbial basal respiration was reduced at lower elevations during the dry season, where soil moisture was low.

Studies in Monteverde have shown that the orographic cloud layer is rising due to the increase in sea surface temperatures, causing an increase in dry days, which are days that receive no precipitation or mist (Pounds et al. 1999, 2006; Still et al. 1999; Lawton et al. 2001; Karmalkar et al. 2008). This trend is especially evident during the dry season and on the Pacific slope of the Cordillera de Tilarán. Ultimately, climate change is exposing this ecosystem to drier and warmer conditions due to the rise of the cloud layer.

Our results suggest that microbial basal respiration could potentially be sensitive to the rising cloud layer. Microbial basal respiration varied with elevation only during the dry season, when clouds typical provide a significant source of water (Holwerda et al. 2010; Goldsmith et al. 2012). Microbial production of CO_2_ might decrease as the cloud layer rises, as microbial basal respiration was particularly high in the sties exposed to cloud cover. The response of microbial production of CO_2_ to changes in temperature and moisture may determine whether TMCF accentuate or mitigate greenhouse gas emissions under future climate.

### Fungal community composition

Our knowledge of how elevation affects belowground communities is limited, and the trends that have been determined are variable, particularly in regards to fungi (Bryant et al. 2008; Meier et al. 2010; Fierer et al. 2011; Bahram et al. 2012; Singh et al. 2012, 2014; Meng et al. 2013; Whitaker et al. 2014b). Most studies have focused on a specific fungal functional group (e.g., Meier et al. 2010; Bahram et al. 2012; Gai et al. 2012; Gómez‐Hernández et al. 2012; Gorzelak et al. 2012; Zimmerman and Vitousek 2012; Coince et al. 2014). This study is one of the first using high‐throughput sequencing of whole soil fungal communities and their responses to changes in elevation, especially within TMCF. Our hypothesis that fungal community composition would shift with elevation was supported. Moreover, we found that fungal communities shifted with the environmental parameters that change with elevation (i.e., temperature, soil moisture content, C:N, pH, and Holdridge life zone). Fungal communities also varied seasonally, but to a lesser extent.

Moreover, we found that taxonomic richness and diversity of fungi declined with elevation according to observed number of fungal OTUs and Simpson's diversity index during the wet season. Others have found declines in richness of plant, animals, and microbes with elevation. This pattern occurs in about 25% of studies, although peaks at mid‐elevations are more common (Rahbek 2005).

As fungal diversity is lower at higher elevations, some combination of dispersal, environmental, and biotic filters may be operating there. In terms of dispersal, winds are often strong in Monteverde, so soil fungi may move along the gradient relatively easily. Regarding environmental filters, soil fungi are sensitive to changes in temperature and moisture (Allison and Treseder 2008; Hawkes et al. 2011; McGuire et al. 2011). Our elevation gradient ranged widely in environmental variables such as soil temperature, moisture content, and C:N over a small distance. These steep changes might have filtered taxa at the higher elevations. However, fungal hyphae were more abundant at higher elevations, and microbial basal respiration did not appear to be inhibited there during the wet season. Thus, we did not detect any evidence that the environmental conditions at the upper sites were particularly stressful for fungi during the wet season, when the declines in richness occurred. It is possible that a biotic filter was acting on fungal taxa. Perhaps competition was stronger at higher elevations, leading to the exclusion of some fungal taxa.

These shifts in fungal community composition were not driven by shifts in the most abundant fungal phyla (i.e., Ascomycota and Basidiomycota). Instead, higher elevations were associated with increases in the number of taxa from the phyla Chytridiomycota and Zygomycota during the wet season. Chytridiomycota and Zygomycota are older phyla that are relatively constrained to regions with higher levels of precipitation (Treseder et al. 2014). An increase in the relative abundance of these phyla with elevation during the wet season may be due to the higher soil moisture content present at high elevation at the end of the gradient.

Functional groups of fungi also responded to elevation, primarily during the wet season. Specifically, the community tended to shift from free‐living filamentous fungi and endophytes at the lower sites toward ECM fungi at the higher sites. For ECM fungi, similar trends have been found along some elevational gradients (Gómez‐Hernández et al. 2012; Miyamoto et al. 2014), but not in others (Kernaghan and Harper 2001; Bahram et al. 2012). Soil conditions can drive richness of ECM fungi, because these fungi can be habitat‐specific (Peay et al. 2010, 2015). For instance, Gómez‐Hernández et al. (2012) reported that soil moisture was related to ECM richness in their elevational gradient. Nitrogen availability might also be a factor. Ectomycorrhizal fungi absorb and deliver soil organic N to plants in exchange for C (Hobbie and Hobbie 2006). Plants may have invested more in ECM fungi at higher elevations, where soil C:N ratios are relatively high and N might limit plant growth. Thus, at higher elevations ECM fungi might be outcompeting free‐living filamentous fungi for C (Gadgil and Gadgil 1971, 1975). If so, this process might act as a biotic filter causing decreased richness at higher elevations.

In contrast, richness of endophytic fungi declined with elevation. Other studies have documented a similar trend (Zimmerman and Vitousek 2012; Ranelli et al. 2015). Endophytes are symbiotic fungi that inhabit the aboveground tissues of plants. Nevertheless, litterfall can deliver endophytic DNA to the soil. Endophytic fungi can confer stress resistance as they reduce plant water loss when water is limiting (Giauque and Hawkes 2013). Endophyte community composition can be driven by rainfall along elevation gradients (Zimmerman and Vitousek 2012). In our gradient, drier conditions at the lower sites could have induced plants to form relationships with more endophytic taxa.

These relationships with elevation – and associated climatic conditions – suggest that fungal communities along this elevation gradient may be vulnerable to climate change. Studies in Monteverde suggest that climate change is exposing this ecosystem to drier and warmer conditions. Our study suggests that both the abiotic (temperature, moisture) and biotic (microbial basal respiration and fungal community composition) properties of soils may be sensitive to the rising cloud layer. CO_2_ emissions might decline based on our observations of basal microbial respiration. Fungal communities may become more diverse, especially in free‐living filamentous fungi. As this functional group includes strong decomposers (Treseder and Lennon 2015), the shift in community composition may mitigate somewhat the decrease in CO_2_ emissions. The balance of these abiotic and biotic responses may determine whether TMCF accentuate or mitigate greenhouse gas emissions under future climate.

Consequently, although this study is observational, it demonstrates that fungal community composition shifts with elevation and with climatic factors that co‐vary with elevation (i.e., temperature and moisture). Moreover, this study suggests that there may be both abiotic and biotic filtering occurring across this elevation gradient. For instance, higher elevations may be suitable environments for fungi, as demonstrated by the higher abundance of fungi and increased microbial basal respiration at these elevations. However, there is decreased richness at higher elevations, suggesting that there is stronger competition occurring at these sites. This was further shown by the differing responses of fungal functional groups to elevation. Ectomycorrhizal fungi may be outcompeting other fungal functional groups, such as free‐living filamentous taxa. Thus, there may be abiotic and biotic mechanisms for decreased rates of decomposition at higher elevations.

### Implications

This elevation gradient can be used to improve our understanding of the roles that temperature and moisture have in influencing belowground communities and processes, and allow for better predictions of how tropical ecosystems will respond to climate change. Studies in Monteverde have shown that the rising cloud layer is particularly influential during the dry season. However, our study demonstrates that there is important structuring of fungal communities during the wet season. Therefore, any changes in climate could have important ecological consequences by potentially changing how these communities are structured, and altering responses of important belowground processes that may mitigate or accentuate climate change. Nevertheless, this study examines soil properties and fungal communities on one elevation gradient over one year, and thus, there is much more to learn within this study system.

## Conflict of Interest

The authors declare no conflict of interest.

## Supporting information


**Table S1.** Field sites locations and life zone assignments along the permanent transect established within the Monteverde Cloud Forest Reserve.
**Table S2.** Changes in soil properties and microbial dynamics with elevation and season.
**Table S3.** Changes in alpha diversity with elevation.
**Table S4.** Relationships of the relative abundance (proportion of sequences) of fungal phyla with elevation.
**Table S5**. Statistics for taxonomic richness of functional groups by elevation and season.
