# Supplementary material for: Belowground responses to elevation in a changing cloud forest
Source: Ecol Evol. 2016 Feb 24;6(7):1996–2009. doi: 10.1002/ece3.2025 (PMC4767876; doi:10.1002/ece3.2025)
Supplement: Supplementary file 1 — Table S1. Field sites locations and life zone assignments along the permanent transect established within the Monteverde Cloud Forest Reserve. Table S2. Changes in soil properties and microbial dynamics with elevation and season. Table S3. Changes in alpha diversity with elevation. Table S4. Relationships of the relative abundance (proportion of sequences) of fungal phyla with elevation. Table S5. Statistics for taxonomic richness of functional groups by elevation and season. [file ECE3-6-1996-s001.docx]

**Supporting Information**

**Table S1.** Field sites locations and life zone assignments along the permanent

transect established within the Monteverde Cloud Forest Reserve.

| **Elevation (m.a.s.l.)** | **Latitude (N)** | **Longitude (W)** | **Holdridge Life Zone** |
| --- | --- | --- | --- |
| 1305 | 10º16'41.71" | 84º47'01.29" | Premontane Forest |
| 1352 | 10º16'41.51" | 84º46'59.27" | Premontane Forest |
| 1399 | 10º17'26.70" | 84º47'34.36" | Premontane Forest |
| 1430 | 10º17'28.81" | 84º47'30.97" | Premontane Forest |
| 1501 | 10º17'43.85" | 84º47'36.96" | Premontane Forest |
| 1549 | 10º18'18.40" | 84º47'46.36" | Lower montane Forest |
| 1600 | 10º18'40.73" | 84º47'56.37" | Lower montane Forest |
| 1656 | 10º18'48.69" | 84º47'57.74" | Lower montane Forest |
| 1698 | 10º18'54.42" | 84º47'52.21" | Lower montane Forest |
| 1743 | 10º18'57.64" | 84º47'47.83" | Montane Forest |
| 1797 | 10º19'01.02" | 84º47'43.80" | Montane Forest |
| 1850 | 10º19'02.02" | 84º47'40.03" | Montane Forest |

**Table S2.** Changes in soil properties and microbial dynamics with elevation and season.^1^

| **Soil characteristics** | **Wet season** | | | **Dry season** | | | **Wet vs. Dry** | |
| --- | --- | --- | --- | --- | --- | --- | --- | --- |
|  | *F* | r^2^ | *p* | *F* | r^2^ | *p* | *F* | *p* |
| Temperature (°C) | 66.5 | 0.861 | **<0.001** | 46.9 | 0.814 | **<0.001** | 35.0 | **<0.001** |
| Moisture (%) | 2.73 | 0.204 | **0.008** | 3.45 | 0.244 | **0.003** | 10.5 | **<0.001** |
| pH | 4.27 | 0.286 | **0.001** | 2.99 | 0.219 | **0.011** | 0.904 | 0.345 |
| Soil C:N | 26.1 | 0.710 | **<0.001** | 5.99 | 0.359 | **<0.001** | 0.548 | 0.462 |
| Microbial basal respiration  (μg CO_2_-C m^-2^ h^-1^) | 0.869 | 0.075 | 0.139 | 10.9 | 0.506 | **<0.001** | N/A | N/A |
| Fungal abundance  (cm m^-2^) | 66.3 | 0.862 | **<0.001** | 18.75 | 0.637 | **<0.001** | 23.4 | **<0.001** |

^1^Significant *p*-values in bold

**Table S3.** Changes in alpha diversity with elevation.^1^

| **Alpha diversity metric** | **Wet season** | | | **Dry season** | | |
| --- | --- | --- | --- | --- | --- | --- |
|  | *F* | r^2^ | *p* | *F* | r^2^ | *p* |
| Observed OTUs | 1.34 | 0.112 | **0.050** | 0.196 | 0.018 | 0.740 |
| Shannon Index | 0.495 | 0.044 | 0.257 | 0.627 | 0.055 | 0.870 |
| Simpson’s Index | 2.87 | 0.212 | **0.008** | 0.738 | 0.065 | 0.255 |

^1^Significant *p*-values in bold.

**Table S4.** Relationships of the relative abundance (proportion of sequences) of

fungal phyla with elevation.^1^

| **Phylum** | **Wet season** | | | **Dry season** | | |
| --- | --- | --- | --- | --- | --- | --- |
|  | *F* | r^2^ | *p* | *F* | r^2^ | *p* |
| Ascomycota | 0.304 | 0.028 | 0.510 | 0.304 | 0.027 | 0.511 |
| Basidiomycota | 0.472 | 0.042 | 0.397 | 0.146 | 0.013 | 0.582 |
| Blastocladiomycota | 1.01 | 0.086 | 0.879 | 1.01 | 0.086 | 0.879 |
| Chytridiomycota | 2.57 | 0.194 | **0.018** | 0.218 | 0.020 | 0.647 |
| Glomeromycota | 0.496 | 0.044 | 0.410 | 7.06 | 0.398 | **0.001** |
| Rozellomycota | 1.24 | 0.104 | 0.078 | 0.342 | 0.030 | 0.501 |
| Zygomycota | 3.56 | 0.250 | **0.003** | 0.712 | 0.062 | 0.429 |

^1^Significant *p*-values in bold.

**Table S5**. Statistics for taxonomic richness of functional groups by elevation and season.^1^

| **Functional group** | **Wet season** | | **Dry season** | |
| --- | --- | --- | --- | --- |
|  | r^2^ | *p* | r^2^ | *p* |
| Free-living filamentous | 0.563 | **0.005** | 0.000 | 0.967 |
| Pathogenic | 0.180 | 0.168 | 0.001 | 0.935 |
| Ectomycorrhizal | 0.542 | **0.006** | 0.284 | 0.074 |
| Yeast | 0.310 | 0.060 | 0.004 | 0.852 |
| Endophytic | 0.565 | **0.005** | 0.035 | 0.561 |
| Lichen | 0.290 | 0.071 | 0.178 | 0.172 |
| Ericoid | 0.063 | 0.432 | 0.018 | 0.679 |
| Arbuscular mycorrhizal | 0.109 | 0.293 | 0.433 | **0.020** |

^1^ Significant *p*-values in bold.
